# Telemetric Assessment of Referred Vaginal Hyperalgesia and the Effect of Indomethacin in a Rat Model of Endometriosis

**DOI:** 10.3389/fphar.2012.00158

**Published:** 2012-08-30

**Authors:** N. Dmitrieva, E. K. Faircloth, S. Pyatok, F. Sacher, V. Patchev

**Affiliations:** ^1^Program in Neuroscience, Florida State UniversityTallahassee, FL, USA; ^2^Bayer HealthCare PharmaceuticalsBerlin, Germany

**Keywords:** analgesia, uterus, prostaglandins, PGE2

## Abstract

Symptoms of endometriosis (ENDO), among others, include pelvic/abdominal and muscle pain. Non-steroidal anti-inflammatory agents are first-line treatment for this pain. Similar to women, rats with surgically induced ENDO, but not its surgical control, exhibit vaginal hyperalgesia, which in rats is evidenced by a decreased threshold for the visceromotor response (VMR) induced by vaginal distention. Here we assess the VMR in rats with implanted probes that telemetrically transmit EMG activity from the abdominal muscle. The feasibility and sensitivity of this technique for monitoring the VMR threshold across the estrous cycle and the influence of Indomethacin on ENDO-induced vaginal hyperalgesia were evaluated. VMR thresholds in response to vaginal distention with an infusion pump were measured in different estrous stages. Indomethacin (5 or 10 mg/kg i.p. or s.c.) was injected in proestrus rats and 40–60 min later the VMR threshold was measured. The VMR threshold varied across the estrous cycle only in ENDO rats, being lowest in proestrus. Indomethacin increased this threshold in proestrus ENDO rats. These results show that telemetric assessment of the VMR is a sensitive tool, suitable for long-term studies in conscious rats. The results with this technique also suggest that ENDO-associated vaginal hyperalgesia involves COX activity, the feature that also underlies inflammatory pains.

## Introduction

Endometriosis (ENDO) is an estrogen-dependent condition defined by the presence of growths of endometrial tissue outside the uterus. ENDO is also considered an inflammatory condition because immune cells and numerous inflammatory mediators including prostaglandins, cytokines, chemokines, and growth factors are involved in its development and maintenance (Bulun et al., 2005; Maybin et al., 2011; Stratton and Berkley, 2011). Among these inflammatory molecules, PGE2 and COX2 appear to play key roles because PGE2 stimulates aromatase activity in endometrial stromal and epithelial cells and thereby promotes local estrogen production that is essential for growth and maintenance of endometrial implants, and COX2 maintains high levels of PGE2 synthesis in ectopic endometrial foci and eutopic endometrium in women with ENDO and itself is subject to regulation by PGE2 and other inflammatory mediators (Noble et al., 1997; Ota et al., 2001; Chishima et al., 2002; Bulun et al., 2005; Banu et al., 2008). In accordance, COX2 inhibitors are effective in reducing ectopic implant growth and improving fertility in murine models when animals are treated in the early stages of ENDO development (Golan et al., 1986; Dogan et al., 2004; Efstathiou et al., 2005; MacHado et al., 2010).

Women with ENDO often experience severe pelvic pain. Non-steroidal anti-inflammatory drugs (NSAIDs) are the first-line treatment for pelvic/abdominal pain but their use is limited because of side effects (Stratton and Berkley, 2011). Surgical removal of lesions often fails to alleviate pain long-term, and hormonal treatments that decrease estrogen levels, although effective for the pain, often have intolerable side effects and are unsafe for long-term use. Therefore, new and long-term treatment options with reduced side effects and efficacy in ENDO are urgently needed.

In an established rat model of ENDO, pieces of uterine tissue are implanted in the abdominal cavity (Vernon and Wilson, 1985). The transplants form cysts that grow rapidly during the first month, continue growing in the following months to reach full size by 8–10 weeks (Vernon and Wilson, 1985). Similar to women, cysts in rats attract their own nerve and blood supply (Zhang et al., 2008; McAllister et al., 2012). Another resemblance to women’s signs is significantly elevated levels of inflammatory mediators (Bergqvist et al., 2001; Anaf et al., 2002; Zhang et al., 2008; MacHado et al., 2010). Importantly like women, rats with ENDO exhibit abnormal sensory signaling associated with the pelvic area, such as vaginal hyperalgesia (dyspareunia in women; Cason et al., 2003) and referred abdominal muscle hyperalgesia (Nagabukuro and Berkley, 2007).

In the previous studies, vaginal hyperalgesia in rats has been assessed either with a behavioral psychophysical test (Cason et al., 2003), or with a technique that measures abdominal muscle electrical activity in response to vaginal distention (Nagabukuro and Berkley, 2007). This later measure, which is a referred abdominal muscle response to noxious stimuli, is called visceromotor response (VMR). Although these techniques are sensitive and have been successfully used to reveal estrous differences in pelvic nociception and effects of drugs and surgical manipulations (Cason et al., 2003; Berkley et al., 2007; Nagabukuro and Berkley, 2007; Dmitrieva et al., 2010), they are labor-intensive and cumbersome to use for drug testing. Here we developed a new technique that combined VMR with vaginal distention and telemetric methodology. We then validated its applicability in two experimental settings. In the first study, we tested changes in ENDO-induced vaginal hyperalgesia across the estrous cycle. In the second study, we tested the hypothesis that COX is involved in pathology of ENDO-associated vaginal hyperalgesia by studying the effect of a non-selective COX inhibitor Indomethacin on the VMR threshold as a proxy of vaginal hyperalgesia.

## Materials and Methods

This study was approved by the Florida State University’s Animal Care and Use Committee, Protocol #0927. The care and use of animals conformed to the recommendations in the Guide for the Care and Use of Laboratory Animals (PHS/NIH) and the Animal Welfare Act (USDA).

### Subjects

Sprague-Dawley female rats were used. Estrous cycle was determined daily by cytological examination of vaginal lavage collected approximately 2 h after lights on (Becker et al., 2005).

*Endometriosis and sham ENDO surgeries* were performed under ketamine/xylazine anesthesia (K/X: 73/8.8 mg/kg i.p.) following aseptic precautions. During surgery and the recovery period, the rat was kept warm on a heating pad. A small incision was made in the middle of the abdomen. A small, approximately 1 cm segment of the middle part of one uterine horn was clamped between two hemostats and excised. The cecum and adjacent intestines were exposed. Four 2 mm × 2 mm biopsies of the excised uterine tissue, or fat in shamENDO rats, were sutured on alternate mesenteric cascade arteries. Muscle and skin were sutured separately. Bupivacaine was given locally and butorphanol, s.c immediately after surgery to alleviate post-operative pain. Rats were monitored on a daily basis for any sign of distress.

### Telemetric probe implantation

Seven weeks after ENDO/shamENDO surgery, under aseptic conditions and K/X anesthesia, a telemetric probe (TA11CTAF40, DSI, St. Paul, MN, USA) was implanted under the skin of the right abdominal flank. Electrodes were tunneled under the skin and implanted in the left inguinal muscle. The skin was sutured. Recovery proceeded as described above. On rare occasions, some rats developed a seroma that was managed by aspiration of clear fluid from the pocket around the probe (under halothane gas anesthesia). This seroma usually subsided in a few days. Rats did not exhibit stress behavior related to the implant. Experiments began at least 7 days after implantation. All rats were habituated to the restraining box and the experimental setting during three to four training sections before VMR recording and did not show any sign of discomfort.

**Figure 1:**
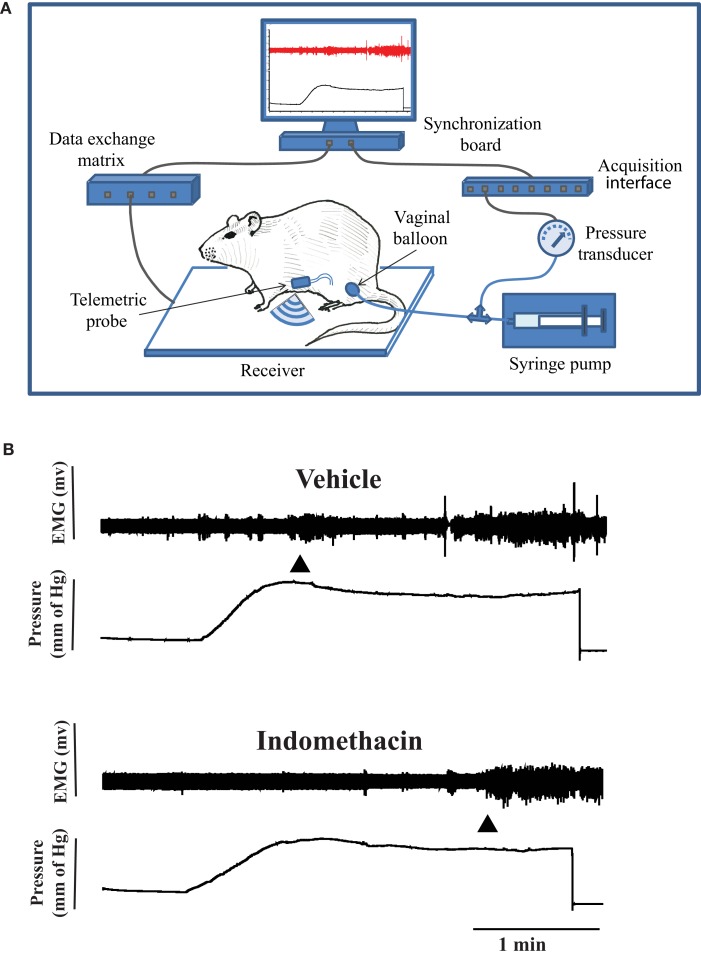
**(A)** Experimental setting for recording of the visceromotor response (VMR) to vaginal stimulation with the Ponemah Telemetric System (DSI, St. Paul, MN, USA). Pressure in the vaginal balloon was generated by an infusion pump. The EMG signal from the electrode implanted in the abdominal muscle was converted into radio signals by the telemetric probe and transmitted to a receiver connected to the acquisition module of the Ponemah System and synchronized with the incoming signal from the pressure transducer. **(B)** A recording of EMG activity during vaginal distension before and after 10 mg/kg Indomethacin (i.p.) injection in the same rat. The first arrow shows the beginning of vaginal distention, and the second arrow shows the VMR threshold. The VMR threshold increased after Indomethacin.

*Visceromotor response* to vaginal distention in conscious rats was assessed with the Ponemah Telemetry System (DSI, St. Paul, MN, USA), which consisted of a receiver, data exchange matrix, acquisition interface, and a computer with a synchronization board (Figure 1A). The rat was placed in a transparent plexiglass box that gently, but not aversively, restrained it. The restraining box was placed on the receiver. A small balloon (∼10 mm diameter when fully inflated) connected to a pressure transducer was inserted into the middle of the vaginal canal. After ∼10 min of resting period, the balloon was inflated by an infusion pump (0.3 ml/min, 1 ml maximum). Electrical activity from the abdominal muscle (electromyography, EMG) was relayed to the DSI system and synchronized with the amplified and digitalized signal from the pressure transducer. EMG activity was recorded during ∼5 min before and during the time when the vaginal balloon was inflated.

The integral of the rectified EMG signal was calculated in 100 ms intervals by the analysis module of the Ponemah System. The volume threshold that corresponded to VMR (Figure 1B) 200% or higher than baseline activity was calculated as reported previously (Nagabukuro and Berkley, 2007). VMR thresholds were obtained 1–2 days between the sessions, two to four baselines per each estrous stage (metestrus DI, diestrus DII, proestrus P, and estrus E,) and then averaged for each stage for each rat.

*Indomethacin* was dissolved in a mixture of DMSO: cremophor: saline (1:1:8). One of two doses (5 mg/kg, i.p. only or 10 mg/kg) or vehicle was injected i.p. or s.c. in the neck area when the rat was in proestrus. The VMR threshold was assessed 40–60 min later.

### Statistical analysis

Differences between VMR thresholds in different estrous stages were analyzed by ANOVA followed by Tukey *post hoc* tests. Differences in VMR thresholds between baseline, vehicle, and Indomethacin groups were analyzed by ANOVA followed by Dunnett or *t*-test as appropriate. Differences with *p* < 0.05 were considered significant.

## Results

### Estrous cycle differences

**Figure 2:**
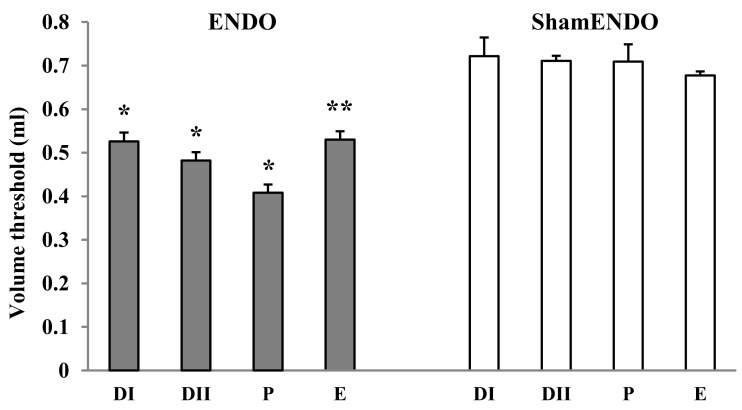
**Estrous variations in VMR to vaginal distention (Volume threshold) in rats >8 weeks after ENDO or shamENDO surgery**. Baseline thresholds were measured across the estrous cycle over 3–5 weeks and then averaged for each estrous stage (i.e., in metestrus DI, diestrus DII, proestrus P, and estrus E). ANOVA showed that thresholds in ENDO rats were significantly different than thresholds in shamENDO rats (*p* < 0.001). *p < 0.005; **p < 0.001, compared to P.

Baseline data were obtained from rats (*n* = 20) in different estrous stages for 3–5 weeks beginning a minimum of 8 weeks after ENDO/sham ENDO surgery when the cyst innervation and vaginal hyperalgesia are fully developed (McAllister et al., 2012). The VMR thresholds were assessed two to four times for each estrous stage for each rat; the individual values remained consistent during the entire study. Average baseline VMR thresholds for each stage are presented for ENDO and shamENDO groups separately in Figure 2.

Statistical analysis showed that VMR thresholds in ENDO rats were significantly different from thresholds measured in shamENDO rats (*p* < 0.001). In agreement with our earlier published data obtained using the VMR technique in lightly anesthetized or tethered rats (Nagabukuro and Berkley, 2007; Dmitrieva et al., 2010), the VMR thresholds varied across the estrous cycle in ENDO rats, with the threshold being lowest in proestrus (*p* < 0.005). On average, the VMR threshold in ENDO rats in the present study in proestrus were 15–19% lower than the thresholds observed in other stages. Again, similar to previous observations, the VMR threshold in proestrus ENDO rats was on average 42.4% lower than that in shamENDO rats. Finally, the threshold in shamENDO rats did not exhibit estrous cycle differences (*p* = 0.752).

**Figure 3:**
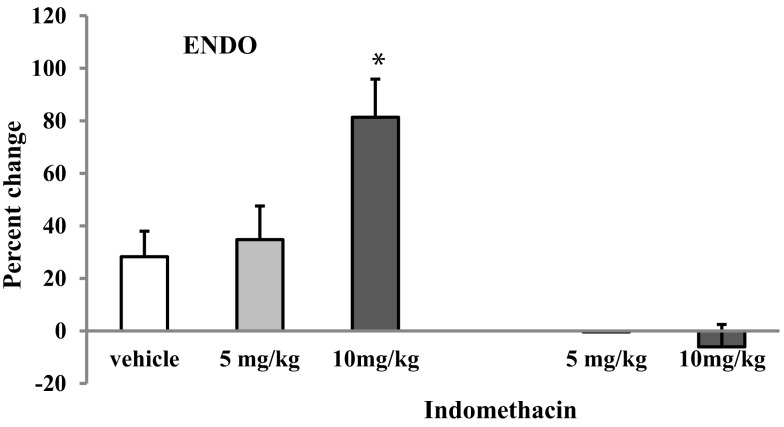
**Effects of i.p. injection of two doses of Indomethacin and vehicle on the VMR threshold in rats after ENDO or Sham ENDO surgery**. Although vehicle produced a small increase in the VMR threshold, this increase was not significantly different from baseline. The increase produced by 10 mg/kg of Indomethacin was significantly higher than the increase produced by vehicle. *p < 0.05 compared to vehicle.

*Indomethacin* was injected first i.p. (5 or 10 mg/kg) in proestrus ENDO and shamENDO rats (*n* = 9). Neither dose of Indomethacin significantly changed the VMR threshold in shamENDO rats. In contrast, the same doses significantly increased the thresholds in ENDO rats in a dose-dependent manner (Figures 1B and 3). The dose 10 mg/kg produced a significant increase in VMR threshold of 81 ± 14.6% compared with both baseline and the effect produced by vehicle (*p* < 0.05).

**Figure 4:**
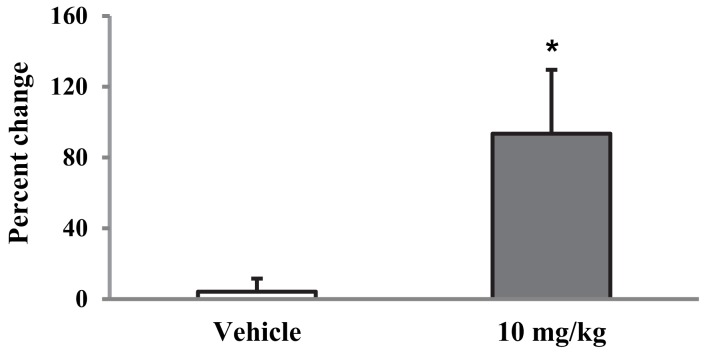
**Effect of 10 mg/kg of Indomethacin injected s.c. in the neck on the VMR threshold**. *p < 0.05 compared to vehicle.

This increase produced by Indomethacin was transient: the threshold measured 4 days later, when the rat was in proestrus, was comparable to baseline values before the treatment (data not shown). Although the vehicle injected i.p. produced a small increase (∼28%, Figure 3), this effect was not significantly different from baseline (*p* = 0.132). To verify whether the vehicle-induced effect was due to the i.p. injection or the vehicle itself, we injected the vehicle and one dose of Indomethacin (10 mg/kg) s.c. in the neck region in a separate group of ENDO rats (*n* = 4, Figure 4). In these rats, Indomethacin but not the vehicle produced a significant increase (*p* < 0.05, 93.5 ± 36%). Therefore, it was the i.p. injection, not the vehicle, that produced the observed increase in the absence of the drug.

## Discussion

Using a telemetric data acquisition technique we were able to carry out a long-term study in conscious rats that, first, involved collecting baseline measures of VMR thresholds across the estrous cycle over 3–5 week period, and then, studying the effect of one or two doses of Indomethacin on the VMR threshold.

The first set of findings confirmed our previously published results on estrous cycle differences in the VMR threshold obtained with a non-telemetered system (Nagabukuro and Berkley, 2007). Similar to this earlier study, ENDO, but not its surgical control (shamENDO), significantly decreased the VMR threshold in all stages of the estrous cycle, being lowest in proestrus (when ovarian hormones plasma levels are highest). Therefore, we confirmed here that in cycling rats surgically induced ENDO produces vaginal hyperalgesia, the severity of which parallels estradiol levels.

In the second set of experiments, we further validated the technique by studying the effect of a NSAID Indomethacin on the hyperalgesia: Indomethacin increased the VMR threshold in ENDO rats when injected either i.p. or s.c. but did not affect this threshold in the shamENDO group: i.e., Indomethacin alleviated only ENDO-induced vaginal hyperalgesia. These findings imply that COX plays a role in maintenance of ENDO-associated vaginal hyperalgesia.

Some possible sites of Indomethacin action include (i) peripheral, locally active COX, whose enzymatic products, such as PGE2, may activate the visceral afferents that innervate the cysts, (ii) COX located in the dorsal root ganglion, and (iii) central sites in the spinal cord and/or brain. Previously, we have shown that afferent fibers, whose branches supply the developed endometrial growths are overactive, which indicates that they are sensitized (Berkley et al., 2004; McAllister et al., 2012). It has been found in other conditions that afferent overactivity can be a result of sensory activation and sensitization by COX2 products (Rendig et al., 1994; Su and Gebhart, 1998; Su et al., 1999).These findings together support a peripheral effect of Indomethacin in the present study. The presence of COX enzymes in dorsal root ganglia and spinal cord, and inhibition of inflammation-induced PGE2 in cerebrospinal fluid and hyperalgesia by intraspinal COX2 inhibitors suggest that Indomethacin could also suppress prostaglandin production centrally (Samad et al., 2001; Yaksh et al., 2001; Dou et al., 2004; Nagabukuro and Berkley, 2007).

The small increase in the VMR threshold that was produced, when the vehicle was injected i.p. was not significant, and was not reproduced after the vehicle was injected s.c. in the neck. This result indicates that the effect was due to the injection itself, not to the vehicle. Thus it is important to recognize that injections may influence the VMR when they are performed close to the recording site (i.e., the abdomen).

Although tethered VMR-based techniques have been proven to be a useful tool in pain-related studies (Nagabukuro and Berkley, 2007; Dmitrieva et al., 2010), frequent repairs of the exposed electrode ends limits its usage in conscious rats to short-term studies. Psychophysics-based technique is an excellent tool for assessing pain-related behavior in rats with ENDO (Cason et al., 2003) but it requires rat pre-screening and training that can limit its applicability when long-term studies are considered. An advantage of telemetry-based VMR technique is that rats require minimal training and are not tethered during the experiment. Together, our findings indicate that the telemetric technique for assessing vaginal hyperalgesia in a rat model of ENDO is feasible and applicable for long-term pharmaceutical studies. Our results also suggest that COX activity plays a role in the vaginal hyperalgesia induced by ENDO. Because products of COX activity can trigger pain in different inflammatory conditions, ENDO-associated pain resembles inflammatory pain. Insofar as findings in the rat relate to ENDO in women, our findings here are in line with other evidence that links COX2 to the development and maintenance of ectopic endometrial growths and together support the conclusion that ENDO-associated pain has inflammatory origins.

## Conflict of Interest Statement

The authors declare that the research was conducted in the absence of any commercial or financial relationships that could be construed as a potential conflict of interest.
